# The Role of Augmented Feedback on Motor Learning: A Systematic Review

**DOI:** 10.7759/cureus.19695

**Published:** 2021-11-18

**Authors:** Arsalan Moinuddin, Ashish Goel, Yashendra Sethi

**Affiliations:** 1 Department of Kinesiology and Sports Management, Texas Tech University, Lubbock, USA; 2 Department of Physiology, Government Doon Medical College, Dehradun, IND; 3 Department of Medicine, Government Doon Medical College, Dehradun, IND

**Keywords:** auditory augmented feedback, multimodal augmented feedback, visual augmented feedback, augmented feedback, motor learning

## Abstract

In motor learning, augmented feedback (AF) is the information provided by sources outside the body and encompasses visual feedback, auditory feedback, and multimodal augmented feedback. This review aims to examine the most recent evidence on these different modality types in healthy and diseased populations and athletes.

The reporting of this review was guided by the standards of the “Preferred Reporting Items for Systematic Reviews and Meta-Analysis (PRISMA)” statement with the aim to examine the most recent evidence on these feedback types in healthy and diseased populations and athletes. The literature search for this review has been limited to electronic journals with the search engines ISI Web of Knowledge, OvidSP EMBASE, and PubMed databases.

This review considers visual feedback as the cornerstone of all augmented feedback types by citing its superiority in learning complex skills by medical students and balance maintenance by older adults. The review also deciphers the role of auditory augmented feedback in encumbering already burdened visual connections in the rehabilitation of patients with Parkinson’s disease (PD) and prosthetic limbs and further argues why the multimodal feedback model seems to be the most efficient way to offer feedback in learning motor tasks by alluding to multifaceted “skill training communication model” in the prevention of sports injuries such as anterior cruciate ligament tears.

Multimodal augmented feedback seems to be the most effective and appropriate way to give feedback during motor learning in healthy and diseased populations and athletes as its stimuli are perceived faster and tend to be retained longer compared with the unimodal stimuli of auditory and visual feedback mechanisms.

## Introduction and background

In motor learning, feedback is movement-related information that is “fed back” to the learner before, during (concurrent), and after (terminal) an attempt to perform a task to enable modifications for the next action. In the field of sports, feedback is given by coaches to athletes to perform better, and in rehabilitation, therapists use it in patients who lost motor functions due to diseases [1,2]. Feedback information can be intrinsic (sensory), if it comes from movement production within the body, and/or extrinsic (augmented), if it is provided by sources outside the body and supplements intrinsic feedback [1]. Our review will exclusively focus on “augmented” feedback (AF), and we will discuss AF studies that looked at motor learning in short-term and long-term retention contexts, both in healthy and diseased populations. The two key variants of AF are knowledge of results (KR), which gives information about the desired outcome (success/failure), and knowledge of performance (KP), which informs the learner about the movement characteristics and its quality [1]. To pragmatically elucidate this difference further, if a golf instructor tells his student that his shot went straight into the right rough, it is KR; however, if he says that the student is short on his backswing, that is KP. AF most often is provided in the form of visual display (screens), auditory stimuli (speakers and headphones), tactile and kinesthetic perception stimuli (haptics), or a hybrid of these modalities. AF research covets to identify the best use of these feedback modalities, keeping these motor learning stages in mind in order to induce enduring changes in motor learning and achieve superior performance. Thus, augmented feedback in motor learning encompasses visual feedback, auditory feedback, and multimodal augmented feedback. The aim of this review was to examine the most recent evidence on these feedback types in healthy and diseased populations and athletes.

Objectives

The current analysis aimed to examine the most recent evidence on different modalities of augmented feedback types (specifically visual, auditory, and multimodal) in healthy and diseased populations and athletes.

## Review

Methods

The reporting of this systematic review was guided by the standards of the Preferred Reporting Items for Systematic Reviews and Meta-Analysis (PRISMA).

Data Sources and Searches

The literature search for this review has been limited to electronic journals with the search engines ISI Web of Knowledge, OvidSP EMBASE, and PubMed databases utilizing the following keywords: (augmented feedback OR visual augmented feedback OR audio augmented feedback OR multi-modal augmented feedback) AND (motor learning).

Article Selection

**Figure 1:**
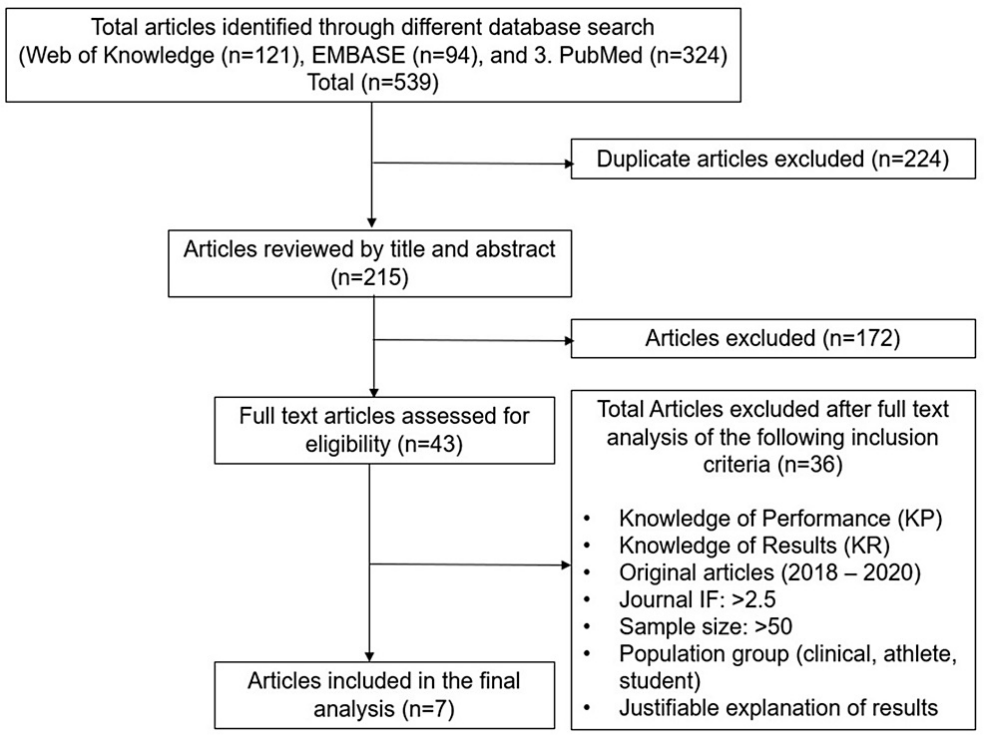
A detailed description of article selection, extraction, and synthesis

The search was limited to English language studies published between 2017 and 2020. A detailed description of article selection, extraction, and synthesis of findings is shown in Figure 1.

Results and discussion

Synthesis of Results and Discussion: Visual Augmented Feedback

Visual augmented feedback is considered the cornerstone of all AF types due to the critical role played by vision as a sensory modality. Although concurrent or real-time visual feedback is vital for learning in the acquisition phase, only a few studies have associated feedback frequencies with motor skill improvement [3,4]. For instance, medical students can profit from concurrent visual feedback while mastering intricate tasks. They learn complex skills during their training period, and they are expected to integrate both practical knowledge and the necessary perceptual-motor skills during their skill training. In a simulation study, Cecilio-Fernandes et al. tried to unveil this paradigm by assigning one of three feedback sources (expert (EF), augmented visual (HS), and expert plus help screen (EF+HS)) to 36 undergraduate medical students who were tested on acquisition and retention of obtaining a transthoracic echocardiogram (TTE) [4]. KR was assessed utilizing the knowledge test and practical skill scores (recorded immediately and after 11 days), whereas KP was evaluated by measuring the time taken to record the images and the quality of the images obtained. At the immediate acquisition stage, they found that the EF group had obtained images faster than the other two groups; however, no group has shown a discernible advantage on speed from a retention viewpoint. In addition, on the image quality work front, the EF+HS group presented superior images than the other two groups [3]. In fact, students benefitted from concurrent visual feedback, and this might be because of the fact that external concurrent feedback plummeted cognitive overload, which in turn enhances overall learning outcomes.

This concurrent visual feedback has its implications in special population groups such as older adults as well. As per the US census projections, the older population is expected to rise from 13% in 2010 to 19% in 2030, with nearly one in five US residents aged 65 and older by then. Aging is associated with loss of muscle mass and strength, which compromises their activities of daily living (ADL) and makes older adults more susceptible to disability, frailty, and fall-related injuries. Activities that involve a one-legged stance are often responsible for trips and falls in older adults due to reduced ability to manipulate sensory information and an overdependence on visual cues. Past evidence suggests that if training methods such as standing on one leg is supplemented by an augmented visual feedback approach, it can enhance both visuomotor processing and balance function. Oungphalachai et al. found improvement in balance ability (static and dynamic) when they provided concurrent customized visual feedback (video screen) to 34 older adults who did slow and sustained single-leg training [5]. Both static balance, measured as “sway velocity,” and dynamic balance, assessed as “directional control of stability in the backward direction,” were improved after training and simultaneous visual feedback, reiterating the significance of temporality and visual signals in augmented feedback learning [4].

Synthesis of Results and Discussion: Auditory Augmented Feedback

Auditory augmented feedback can play a major role in the disease rehabilitation process because recovery strategies are heavily based on cognitive grasping methods that mainly focus on response to visual stimuli [5]. As a consequence, supplemental visual AF tends to encumber already burdened visual connections. Thus, auditory AF provides a fresh perspective to the learner in terms of feedback and improves overall learning by reducing subjective workload [5]. This was recently demonstrated by Carvalho et al. who incorporated auditory AF in a new strategy employed by physical therapists to mitigate Parkinson’s disease (PD) gait symptoms by “placing the heel first when stepping” [2]. PD is a neurodegenerative disorder that affects around 1% of the old age population, and approximately 20% of these patients have gait disturbances. Gait in PD typically manifests as stooped posture with quick, short, and shuffling steps, with an overall lack of steadiness and smoothness in step progression that leads to frequent trips and falls. Gait training in PD requires motor skill development, which is tedious and takes time before it becomes automatized and consistent. Carvalho et al. devised a Heel2Toe sensor device that gives real-time auditory feedback to PD gait rehabilitation patients for each step they take that reaches an appropriate heel strike angle. Their hypothesis emphasized that real-time feedback is critical for PD gait rehabilitation because “closer feedback to the time of action” facilitates the learning process. Their strategy seemed potentially effective as their participants who received five sessions of training for two to three weeks exhibited greater audiovisual scores, enhanced percentage of good steps, and homogeneous cadence, although a small sample size of six participants sometimes raises questions about the validity of such results [6]. The work of Carvalho et al. was based on the “auditory alarm” variant of auditory AF, which produces sound without tone modulation when the movement variable exceeds the allocated threshold. For instance, the alarm rings when it senses unequal activity or loading of the weakened limb as compared with its normal counterpart [2].

In recent years, prosthetic science also employed auditory AF in an attempt to make prosthetics more efficient and user-friendly. It is extremely challenging for upper limb amputees with prosthetic devices to incorporate their previous knowledge and sensory feedback information (tactile, auditory, and visual) to accomplish motor tasks, unlike their normal counterparts, because they rely heavily on visual feedback. This overdependence on visual feedback is in part due to loss of tactile feedback, as well as expending more time in watching how prosthetics work. Thus, to combat this extra reliance on vision and enhance the clinical usability of hand prosthetic devices, Shehata et al. suggested the use of an auditory augmented real-time feedback system when controlling a prosthetic hand. They investigated the feasibility of this feedback modality in 14 able-bodied subjects through a system of headphones coupled with a test object (fitted with force sensors). For both psychophysical and performance tests, they found identical improvements in task acquisition ability [7]. However, the model design of this study compelled researchers to limit their results to short-term performance only.

Synthesis of Results and Discussion: Multimodal Augmented Feedback

Solitary visual AF or auditory AF given alongside the motor task tends to improve learning a bit, but their individual efficacies seem low as compared with their concomitant combined effect as the human brain is designed to process information much better and faster if the feedback comes from a variety of sources simultaneously. For example, if we listen to sports broadcast on radio (auditory perception only) or see it on a muted TV (visual perception only), in each case, we tend to concentrate harder, and still, the experience is not of optimum quality, in comparison to watching it on a TV with the sound on (both auditory and visual), which will give the best experience with less effort. Thus, it is perceived that the augmented multimodal feedback model is the most efficient means of offering feedback in learning motor tasks.

To impugn the debate that traditional athletic coaches overuse verbal feedback to help trainees perform specific movements optimally, Otte et al. proposed a multifaceted “skill training communication model” that includes a variety of athlete-specific feedback methods, such as task-oriented question-answer (Q-A) approach, trial approach, analogy learning, and live video feedback. This model encourages coaches to modify their roles more as “moderators” and/or “facilitators” rather than adopting a conventional didactic approach. This approach aimed to consider a series of feedback and instructional methods that individual athletes want and respond the best to ensure the best performance outcome as compared with traditional pedagogical methods that often impinge athletes to perform to their maximal ability [8].

Multimodal AF is employed in the prevention of sports injuries as well. An anterior cruciate ligament (ACL) tear is one particular injury that affects nearly 250,000 physically active young adult Americans (especially athletes) each year. High treatment costs and lengthy rehabilitative phases pose a substantial fiscal burden on the individual and the economy as a whole. A vast majority of these injuries happen due to inappropriate jump-landing techniques and can be prevented if proper motor learning is provided in the form of augmented feedback information to these athletes. To elucidate the role of AF in ACL injuries, Armitano et al. undertook this systematic review to examine coherence in different studies that evaluated various AF methods and their combinations to identify safe jump-landing techniques that mitigate the risk of ACL injury. The authors searched four electronic databases to include 18 studies in their final analysis and came up with some encouraging results. They found augmented prescriptive information alone helpful in reducing the peak vertical ground reaction forces (GRFs) with the verbal cues, such as “knees over toes” (internal focus) and “land softly” (external focus), and improvement in terms of kinematic and kinetic factors in athletes who were provided visual augmented feedback during landing. However, a combination of prescriptive and combined feedback techniques proved most effective and exhibited large effect sizes for knee, hip, and trunk flexion angle, suggesting a maximal reduction in vertical GRFs and thus offering beneficial injury prevention approaches in terms of the mingling of feedback methods [9].

Limitations

The major limitations of this review are the choice of only a few most recent articles (2017-2020) out of many available on this topic and alluding synthesis of findings and discussion to only key modalities of AF in motor learning.

## Conclusions

Together, multimodal augmented feedback seems to be the most effective and appropriate way to give feedback during motor learning in healthy and diseased populations and athletes. Multimodal stimuli are perceived faster and tend to be retained longer than unimodal stimuli, which somewhat justify the adequacy and competency of multimodal AF methods. Their additive effect can be explained based on sensory enhancement or intersensory facilitation theory by Carson who postulated that once information is mediated by many stimuli simultaneously, there is an increased engagement of anterior cingulate cortex (ACC) in the brain that stabilizes AF signal quicker and entrench them deep in the brain. The reason is further perceived as a potential cross talk between the signals that potentiates the overall response.
